# Development and Validation of a Rapid Point-of-Care *CYP2C19* Genotyping Platform

**DOI:** 10.1016/j.jmoldx.2024.12.001

**Published:** 2024-12-24

**Authors:** Kerry A. Burke, James O'Sullivan, Nicola Godfrey, Videha Sharma, Sian Hilton, Stuart J. Wright, Nicholas S. Greaves, William G. Newman, John H. McDermott

**Affiliations:** ∗ Manchester Centre for Genomic Medicine, St Mary's Hospital, Manchester University NHS Foundation Trust, Manchester, United Kingdom; † Division of Evolution, Infection and Genomics, School of Biological Sciences, University of Manchester, Manchester, United Kingdom; ‡ Manchester Vascular Centre, Manchester Royal Infirmary, Manchester University NHS Foundation Trust, Manchester, United Kingdom; § Manchester Centre for Health Economics, University of Manchester, Manchester, United Kingdom

## Abstract

Pharmacogenetic-guided prescribing can lead to more accurate medicine selection and dosing, improving patient outcomes and leading to better use of health care budgets. Loss-of-function variants in *CYP2C19* influence an individual's ability to metabolize clopidogrel, increasing the risk of secondary vascular events following ischemic stroke and percutaneous coronary intervention. In acute clinical contexts, centralized laboratory-based testing is too slow to inform timely clinical decision-making. This work reports the development and analytical validation of the Genedrive *CYP2C19* ID Kit, which provides rapid point-of-care genotyping from a buccal swab in approximately 1 hour. Buccal samples were collected from a total of 204 individuals between September 2023 and July 2024, alongside a blood or saliva sample for comparison with laboratory testing. In the final cohort of 202 patients, all point-of-care results were concordant with laboratory testing. In this assessment, the sensitivity and specificity of the *CYP2C19* ID Kit was 100% (95% CI, 95.0%–100%) and 100% (95% CI, 97.2%–100%), respectively. The failure rate of the *CYP2C19* ID Kit was 0.98%. This study confirms the analytical validity of the Genedrive *CYP2C19* ID Kit. The Genedrive system is able to provide an accurate, rapid, noninvasive alternative to standard laboratory testing and can be used as a point-of-care test in the clinical environment.

An individual's genetic profile can be used to select personalized and effective medicines to optimize patient outcomes, a concept known as pharmacogenetics. Traditionally, genetic testing has been undertaken using laboratory-based techniques, which are lengthy and logistically complex processes requiring blood or saliva specimens to be sent to diagnostic laboratories, which may be located far from the point of clinical need. In the context of genetic testing for rare inherited disease, which has historically represented most of genomic laboratory activity, this testing approach has not been problematic as results are rarely required rapidly to inform treatment decisions. However, the emergence of pharmacogenetics as a clinical entity has led to scenarios where results are required to guide treatment in the acute clinical setting.^1^ To address this unmet need, point-of-care testing devices have been developed, which are able to provide rapid patient genotype results for the management of time-critical conditions.

One such time-critical condition is neonatal sepsis, where aminoglycosides are indicated within 1 hour after diagnosis.^2^ The *MT-RNR1* m.1555A>G variant is known to predispose to severe and irreversible aminoglycoside-induced ototoxicity after just a single dose.^3^ In this acute scenario, standard laboratory-based techniques were not viable. As such, a rapid point-of-care test (POCT) was developed by Genedrive, which could test for the m.1555A>G variant in just 26 minutes. This was integrated into the admission pathways at two neonatal intensive care units, and the results were able to successfully guide antibiotic prescribing without disrupting normal clinical pathways.^2^ This testing strategy has since been incorporated into national guidelines in the United Kingdom through the National Network for Health and Care Excellence (NICE; Health Technology Evaluation 6, *https://www.nice.org.uk/guidance/hte6*, last accessed November 6, 2024).

More recently, NICE has published diagnostic guidance recommending *CYP2C19* genotyping in patients to guide treatment following ischemic stroke and transient ischemic attack (TIA; Diagnostic Guidance 59, *https://www.nice.org.uk/guidance/dg59*, last accessed November 6, 2024). Furthermore, the American Heart Association has produced a scientific statement to outline its position that the evidence to date supports *CYP2C19* genetic testing before oral P2Y12 inhibitors are prescribed in patients with acute coronary syndrome or percutaneous coronary intervention.^4^ The CYP2C19 enzyme metabolises the thienopyridine prodrug clopidogrel, the active metabolite of which selectively and irreversibly inhibits platelet aggregation. Loss-of-function (LoF) variants in the *CYP2C19* gene are frequent in the population, with >35 known allele haplotypes.^5^ The ∗2, ∗3, ∗4, ∗8, and ∗35 alleles are examples of LoF alleles.^5^ Conversely, the ∗17 allele results in *CYP2C19* gain of function, increasing enzyme activity. The wild-type (∗1) allele has normal function and reflects a result where no loss or gain of function variants have been identified. Humans have two *CYP2C19* alleles, and the combination of these alleles in diplotype defines an individual's CYP2C19 metabolizer state. The presence of one LoF allele, in combination with a normal function (∗1) allele, results in intermediate metabolizer status (eg, ∗1/∗2), and two LoF alleles results in poor metabolizer status (eg, ∗2/∗3) (Table 1).

**Table 1:** *CYP2C19* Alleles and Metabolizer Status

Diplotype	Allele 1						
	∗1	∗2 (LoF)	∗3 (LoF)	∗4 (LoF)	∗8 (LoF)	∗17 (GoF)	∗35 (LoF)
Allele 2							
∗1	NM	IM	IM	IM	IM	RM	IM
∗2 (LoF)	IM	PM	PM	PM	PM	IM	PM
∗3 (LoF)	IM	PM	PM	PM	PM	IM	PM
∗4 (LoF)	IM	PM	PM	PM	PM	IM	PM
∗8 (LoF)	IM	PM	PM	PM	PM	IM	PM
∗17 (GoF)	RM	IM	IM	IM	IM	URM	IM
∗35 (LoF)	IM	PM	PM	PM	PM	IM	PM

An individual's CYP2C19 metabolizer state is predicted by his/her *CYP2C19* star allele (∗) diplotype.

GoF, gain-of-function allele; IM, intermediate metabolizer; LoF, loss-of-function allele; NM, normal metabolizer; PM, poor metabolizer; RM, rapid metabolizer; URM, ultrarapid metabolizer.

Carriers of *CYP2C19* LoF alleles are at a significantly increased risk of secondary vascular events following percutaneous coronary intervention, and ischemic stroke or TIAs.^6^, ^7^, ^8^, ^9^, ^10^, ^11^ In these patient groups, genotype-guided prescribing has been shown to be a cost-effective strategy, and some health systems have introduced *CYP2C19* genotyping into routine practice.^12^, ^13^, ^14^ If adopted routinely, the most recent recommendations from NICE would result in approximately 100,000 additional genetic tests each year in the United Kingdom given the incidence of ischemic stroke and TIA.^15^ At present, *CYP2C19* genotyping is not widely available from the genetic laboratories that serve the UK National Health Service (NHS) and, as existing infrastructure has been developed to mainly deliver rare disease and cancer testing in the outpatient context, results may take many weeks. Given the potential scale of *CYP2C19* testing recommended by NICE and considering the acute nature of most stroke admissions, with a clear time pressure to commence definitive antiplatelet therapy, this represents a major unmet need.

A POCT approach could address this challenge, with the capability to generate results in a clinically relevant time frame without the need to send samples to centralized laboratories. This article reports on the development and analytical validation of the Genedrive *CYP2C19* ID Kit, a technology designed to address this significant unmet need by generating results in a clinically relevant format and time frame at the point of need.

## Materials and Methods

### Subject Enrollment and Participation

This analytical validation study of the Genedrive *CYP2C19* ID Kit was undertaken as a subanalysis of the Implementing Pharmacogenetics to Improve Prescribing trial (ISRCTN14050335), a prospective observational cross-sectional design. Participants were recruited from two sites at the Manchester University NHS Foundation Trust in the United Kingdom. Participants were either recruited as outpatients from the Manchester Centre for Genomic Medicine or as inpatients from the Manchester Royal Infirmary. Participants consented to a single venous blood or saliva sample (at their preference), as well as a buccal swab.

### Ethical Approval

The study adhered to good clinical practice guidelines and the Declaration of Helsinki. All participants provided written informed consent before participation, and the study received approval from the NHS research ethics committee (IRAS 305751) and the Human Research Authority.

### The Genedrive *CYP2C19* ID Kit

**Supplemental Figure S1:**
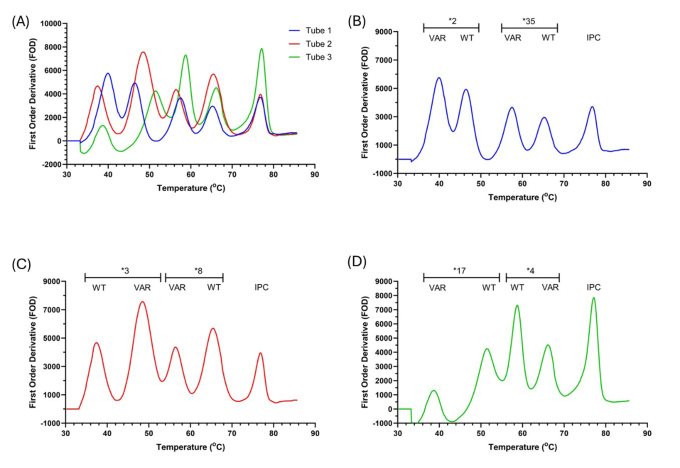
Melt curve analysis. **A:** All possible melt profiles across three tubes. **B:** Possible melt profiles in tube 1 (∗2 and ∗35). **C:** Possible melt profiles in tube 2 (∗3 and ∗8). **D:** Possible melt profiles in tube 3 (∗17 and ∗4). IPC, internal positive control; VAR, variant allele; WT, wild type.

The Genedrive platform is a portable PCR thermocycler that performs real-time nucleic acid amplification and end-point melt analysis detection. The *CYP2C19* ID Kit, designed to operate on the Genedrive platform, has been designed to detect the ∗2, ∗3, ∗4, ∗8, ∗17, and ∗35 *CYP2C19* alleles (Supplemental Figure S1) from a buccal swab. Detailed information on the laboratory preclinical validation of the test is provided in the manufacturer's instructions for use (Genedrive PLC, Manchester, UK).

The Genedrive *CYP2C19* ID Kit instructions for use, which was followed throughout this study, recommend that the buccal swab is rubbed across the patient's cheek 10 times on each side before submerging the tip into the kit buffer solution and rotating the swab for 30 seconds. Following PCR amplification, end-point melt curve detection occurs, and an automated fluorescence signal processing algorithm on the Genedrive platform then reports both diplotype and the metabolizer state, based on Clinical Pharmacogenetic Implementation Consortium guidance.^5^ The result can then be presented to the user via a touch screen and can be securely messaged to integrate with electronic health records. Where the genedrive instrument is unable to determine the *CYP2C19* result, a test fail outcome is produced. If no DNA is detected, suggesting the buccal swab did not collect sufficient material, the instrument will inform the user no human DNA detected.

Buccal samples were collected and tested via the Genedrive *CYP2C19* ID Kit by patient-facing health care professionals who underwent a 2-hour training session. Genedrive instruments were located in the Manchester Centre for Genomic Medicine, where testing took place. The Genedrive assay cartridge contains the reagents needed to perform amplification and detection, in a lyophilized format stored at room temperature. The lyophilized reagents are reconstituted with the lysis buffer using a transfer capillary. The assay cartridge is then inserted into the Genedrive system, and the testing process takes 69 minutes. The results from the Genedrive *CYP2C19* ID Kit were recorded in a secure database with a study ID by a member of the trial team. Preclinical performance data, including assessment exclusivity and the impact of interfering substances, can be found in the instructions for use (Genedrive PLC).

### Reference Test

In an ISO15189 accredited laboratory, DNA was extracted from the blood or saliva samples and then genotyped using the Agena Veridose Core V1.0 panel (Agena Bioscience, San Diego, CA), which is the current clinical reference genotyping approach used in the NHS Northwest Genomic Laboratory Hub. The Veridose Core V1.0 panel tests for all alleles included in the Genedrive *CYP2C19* ID Kit, except for the ∗35 allele (Supplemental Table S1). A full list of *CYP2C19* alleles included in the Veridose Core V1.0 panel is provided in Supplemental Table S1. Results of the Genedrive *CYP2C19* ID Kit were directly compared with the Agena results. Where the Agena system was unable to produce a result despite adequate DNA concentration and quality, this was recorded as test fail.

Although the Agena Veridose Core V1.0 represents the most frequently used pharmacogenetic test in the reference laboratory, having undergone local verification, it is designated by the manufacturer as for research use only. As such, any discordant results between the Genedrive and Agena platforms, or any test fails, were genotyped via Sanger sequencing, representing a gold standard testing approach. Sanger sequencing primers were designed to detect the ∗2, ∗3, ∗4, ∗8, ∗17, and ∗35 alleles (Table 2).

**Table 2:** Sanger Probe Sequences

CYP2C19 Sanger probes	Sequence
Exon 1 forward	5′-TAGTGGGCCTAGGTGATTGG-3′
Exon 1 reverse	5′-CTCCATGTTACAATGTTCTCTAGTAAC-3′
Exon 2-3 forward	5′-TTTGAGCCTGTGTGACTGAA-3′
Exon 2-3 reverse	5′-AGAACCTTTGTAAAGTCCCC-3′
Exon 4 forward	5′-TTTGCTTTTAAGGGAATTCATAGGT-3′
Exon 4 reverse	5′-GATCTGTCGGTACCCCACTT-3′
Exon 5 forward	5′-TCTCTTGTCAGAATTTTCTTTCTCAA-3′
Exon 5 reverse	5′-ACACTGACGAACGCATAAACAC-3′
Exon 6 forward	5′-CCCTCTCTCACCGCTCCTAT-3′
Exon 6 reverse	5′-ACGACGATCACAAGAGGAAAGA-3′
Exon 7 forward	5′-TTCATGTACCCCTGAATTGCT-3′
Exon 7 reverse	5′-GTGACCCACTCTCTTCACGT-3′
Exon 8 forward	5′-TGCATGATTACCACTGTTTCTTA-3′
Exon 8 reverse	5′-TGAATGTACACGGAAGAGACGT-3′
Exon 9 forward	5′-TCACCGAACAGTTCTTGCAT-3′
Exon 9 reverse	5′-CGAAGAAGACTGGGCAGTAG-3′
∗17 Forward	5′-ATCTCTGGGGCTGTTTTCCT-3′
∗17 Reverse	5′-TTGTTAAGGACGGAAGTGCA-3′

### Statistical Analysis

The study was powered to achieve a diagnostic performance of >99% sensitivity and specificity, for the detection of the prespecified *CYP2C19* alleles, with a CI of >95%. This assumed an expected *CYP2C19* allele frequency of 0.3696 in the population, based on reported frequencies in the European population.^5^ Accounting for Hardy-Weinberg equilibrium, this estimates an allele carrier prevalence of 0.602 within the European population. As such, to achieve the desired performance, testing samples from at least 200 individuals was required. The 95% CIs are provided for sensitivity, specificity, and accuracy. These are exact Clopper-Pearson CIs. A subanalysis of diagnostic accuracy for the detection of gain-of-function and LoF alleles was also undertaken.

## Results

### Analytical Performance

Samples were collected from a total of 204 adults between September 1, 2023, and July 1, 2024. Of the 204 samples collected, a result was achieved with the Genedrive *CYP2C19* ID Kit for 202 (99.02%). One sample was reported as a test fail, and another was reported as no human DNA detected. These represent a failure of the end-to-end testing process, resulting in a first test failure rate of 0.98% across the assessment.

**Figure 1:**
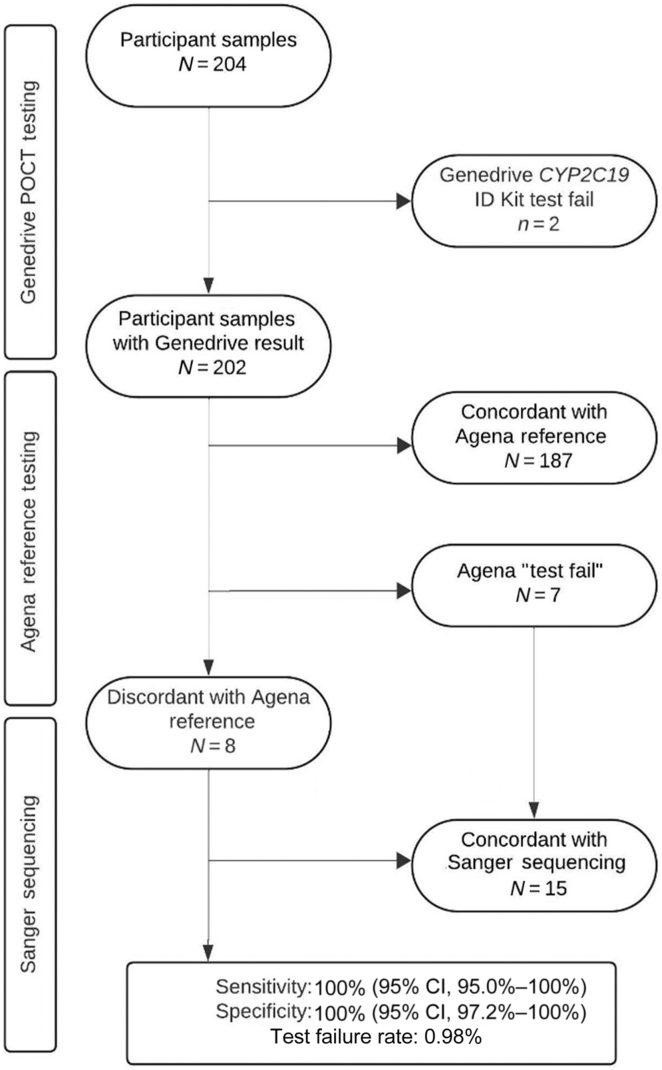
Results flow diagram. Samples were tested via the Genedrive *CYP2C19* ID Kit, before clinical reference testing via the Agena Veridose Core V1.0 panel (Agena Bioscience, San Diego, CA). Any discordant or failed results from the Agena testing were sequenced via gold standard Sanger sequencing. POCT, point-of-care test.

Of the 202 remaining samples, results from the Genedrive *CYP2C19* ID Kit were 100% concordant with the eventual reference standard (Figure 1). Testing via the Agena Veridose Core V1.0 panel produced seven test fails (3.5%), and eight results were discordant with the Genedrive *CYP2C19* ID Kit. Testing of those 15 samples via Sanger sequencing found that the Genedrive *CYP2C19* ID Kit was accurate in each case. In total, 72 patient samples tested positive for a clinically relevant (ie, non ∗1) allele, whereas the overall sensitivity and specificity of the Genedrive *CYP2C19* ID Kit for all clinically relevant alleles was 100% (95% CI, 95.0%–100%) and 100% (95% CI, 97.2%–100%), respectively. The sensitivity and specificity for *CYP2C19* LoF alleles was 100% (95% CI, 94.0%–100%) and 100% (95% CI, 97.4%–100%), respectively, and for the *CYP2C19∗17* gain-of-function allele, they were 100% (95% CI, 94.7%–100%) and 100% (95% CI, 97.2%–100%). The complete data set is provided in Supplemental Table S2.

### Discordant Results

Eight participants were discordant between the Genedrive *CYP2C19* ID Kit and the Agena Veridose Core V1.0 panel. Orthogonal Sanger sequencing showed the Genedrive result to be accurate in all nonconcordant cases. Three of these discordances appeared to be produced as the MassArray Analyzer, and its automated typing software, did not detect sufficiently large peaks to indicate the presence of a specific allele. One discordance occurred as the MassArray Analyzer erroneously identified a peak that corresponds to the ∗17 allele, which was in fact not present. Three discordant results occurred because the Agena Veridose Core V1.0 panel does not detect the *CYP2C19 ∗35* LoF allele, whereas the Genedrive *CYP2C19* ID Kit can. No additional rare alleles included on the Agena Veridose Core V1.0 panel, and not included on in the Genedrive *CYP2C19* ID Kit, were detected as part of this assessment.

A final discordant sample had a Genedrive *CYP2C19* ID Kit result of ∗1/∗17 and an Agena result of ∗1/∗1. On review, it became apparent that this patient had undergone a hematopoietic stem cell transplant. As the Agena genotyping test had been performed on DNA extracted from a blood sample, it was hypothesized that the discordance derived from the donor transplant cells. The buccal sample was therefore tested using Sanger sequencing, as this would reflect the host-derived sample. This confirmed a concordant ∗1/∗17 result with the POCT.

## Discussion

This validation study of the Genedrive *CYP2C19* ID Kit demonstrates that this rapid point-of-care assay has an analytical performance with a sensitivity and specificity of 100% (95% CI, 95.0%–100% and 97.2%–100%, respectively). During this assessment, the Genedrive *CYP2C19* ID Kit performed better than the widely used laboratory reference test, with a superior accuracy and a lower failure rate. This robust analytical performance, coupled with the fact that a result can be generated at the point of care in approximately 1 hour, means the system has several distinct advantages over existing laboratory-based and comparator POCT genetic testing approaches.

Following an ischemic stroke or TIA, the risk of secondary events is highest in the first 90 days and decreases thereafter.^16^^,^^17^ The risk of a further stroke within the first week is substantial, highlighting the need for prompt and effective secondary prevention.^16^ The prescription of an antiplatelet is a key aspect of this management. National and international guidance now recommends that *CYP2C19* genotyping should be used to inform antiplatelet prescribing practice.^4^^,^^5^^,^^18^ In many health systems, including the NHS in England, most genetic testing is currently undertaken in centralized laboratories. Most stroke centers will not be colocated alongside these laboratories, meaning any sample for *CYP2C19* testing will need to be transported to a separate health care organization for testing, introducing significant logistical complexity and time burden.

Once the sample has been tested, the result then needs to be returned to the health care professional who can use the CYP2C19 status to guide prescription. However, for minor strokes and TIAs, the mean length of hospitalization is approximately 2 to 3 days, varying depending on geography and clinical context. As such, even with an optimized laboratory process, in many cases results will be returned after the patient has left the hospital, necessitating the development of new clinical pathways to recall the patient and potentially alter his or her prescription. This introduces further complexity, delays, and costs. Integrating genetic laboratory testing into an acute clinical pathway, such as for ischemic stroke or percutaneous coronary intervention, would be highly complex, have multiple points of failure, heavily rely on human input, and thus require significant investment to deliver an equitable and scalable service. A POCT approach, as delivered by the Genedrive *CYP2C19* ID Kit, dispenses with the need to send samples to a centralized laboratory and ensures that results are available at the point of need.

The recent diagnostics guidance from NICE (Diagnostic Guidance 59) recommended laboratory testing, where available, over a POCT approach in part because laboratory-based tests could theoretically test for a broader range of LoF alleles, and so would better serve ethnic minority groups who are more likely to carry these rarer variants. The Genedrive *CYP2C19* ID Kit genotypes all of the *CYP2C19* tier 1 variant alleles, as defined by the Association for Molecular Pathology, and several of the *CYP2C19* tier 2 variant alleles.^19^ Those tier 2 alleles not tested (∗5, ∗6, ∗7, ∗9, and ∗10) represent a combined allele frequency of 0.1%–2.6%, depending on the biogeographical group. This represents relatively comprehensive coverage of currently known *CYP2C19* variation and, by being able to test for the ∗35 allele that is present in 3.2% of sub-Saharan Africans and 1.6% of African Americans/Afro-Caribbeans, the Genedrive *CYP2C19* ID Kit is therefore able to target a broader set of alleles than many laboratory-based platforms.^5^^,^^19^

The assertion that, in theory, laboratory-based testing approaches allow a broader coverage of alleles is accurate. However, laboratory testing is heterogeneous and, in practice, most *CYP2C19* testing approaches used in practice remain genotyping based and survey a small number of clinically relevant variants. More detailed sequencing-based approaches to include rarer, less well-evidenced, variants are available but come with several trade-offs. The cost, complexity, and turnaround time of these approaches would require considerable adaptation to existing clinical pathways, and there is significant uncertainty regarding whether they could be successfully implemented in the context of an acute presentation, such as ischemic stroke. These technologies may add value when used pre-emptively, where pharmacogenetic data are integrated into a patient's clinical record, but they are not easily conducive to a reactive implementation model. As part of their assessment, NICE found use of *CYP2C19* genotyping to guide antiplatelet therapy was likely to be cost-effective via both laboratory and POCT approaches, including with the Genedrive *CYP2C19* ID Kit.

The Genedrive *CYP2C19* ID Kit compared favorably to the reference laboratory test in this assessment, and it has several advantages over the existing POCT on the market. The Genomadix Cube *CYP2C19* POCT (Genomadix, Kanata, ON, Canada) has been extensively tested and is able to provide rapid and accurate *CYP2C19* genotype data.^20^, ^21^, ^22^ However, as currently designed, the technology can only detect the ∗2, ∗3, and ∗17 alleles, has a higher reported price point, and, critically, requires cold reagent storage. In many acute clinical settings, freezer storage may not be available and, even if it is, having to consider this in the testing pathway introduces a cognitive burden and an implementation barrier. The lyophilized reagents used by the *CYP2C19* ID Kit avoids this. The Genedrive technology has previously been implemented in the neonatal intensive care unit, a hyperacute setting, to avoid aminoglycoside-induced hearing loss.^2^ The fact that this has been implemented successfully, requiring minimal training, demonstrates the ability of this platform to provide genetics at the point of need, democratizing access to pharmacogenetic-guided therapy.

## Conclusion

This validation study demonstrates the Genedrive *CYP2C19* ID Kit to have a high diagnostic accuracy for clinically relevant *CYP2C19* variation. The platform had a superior performance to the reference test, with a higher accuracy and lower failure rate. Implementation of the Genedrive *CYP2C19* ID Kit in clinical practice could provide an accurate, rapid, cost-effective, and noninvasive alternative to standard laboratory testing and ensures results are available in a clinically relevant format and time frame to guide patient care.

## Disclosure Statement

J.H.M., V.S., and W.G.N. are cofounders of Fava Health, a health technology consultancy. Genedrive was one of several industry coapplicants with the authors on the Innovate UK-funded Development and Validation of Technology for Time Critical Genomic Testing Programme (10058536), which supported this work.
