# Supplementary material for: Development and Validation of a Rapid Point-of-Care CYP2C19 Genotyping Platform
Source: J Mol Diagn. 2024 Dec 24;27(3):209–15. doi: 10.1016/j.jmoldx.2024.12.001 (PMC12179497; doi:10.1016/j.jmoldx.2024.12.001)
Supplement: Supplemental Table S1 [file mmc1.docx]

| **Gene** | **Nucleotide Change** | **Amino Acid Change / Star Allele** | **dbSNP RS#** |
| --- | --- | --- | --- |
| *CYP2C19* | c.681G>A | *2 | rs4244285 |
|  | c.636G>A | *3 | rs4986893 |
|  | c.1A>G | *4 | rs28399504 |
|  | c.1297C>T | *5 | rs56337013 |
|  | c.395G>A | *6 | rs72552267 |
|  | g.19294T>A | *7 | rs72558186 |
|  | c.358T>C | *8 | rs41291556 |
|  | g.-806C>T | *17 | rs12248560 |

**Supplemental Table S1.** *CYP2C19* alleles tested on the Agena VeriDose Core V1.0 Panel. Variants and dbSNP RS numbers were accessed on 8 August 2024 from the Agena Bio Website (Agena Bioscience, San Diego, USA). The *CYP2C19* variants can be viewed using the dbSNP RS# via <https://www.ncbi.nlm.nih.gov/snp> (last accessed December 11, 2024).
