# Supplementary material for: Development and Validation of a Rapid Point-of-Care CYP2C19 Genotyping Platform
Source: J Mol Diagn. 2024 Dec 24;27(3):209–15. doi: 10.1016/j.jmoldx.2024.12.001 (PMC12179497; doi:10.1016/j.jmoldx.2024.12.001)
Supplement: Supplemental Table S2 [file mmc2.docx]

| **Study ID** | **Genedrive *CYP2C19* diplotype** | **Agena MassARRAY**  ***CYP2C19* diplotype** | **Sangar Sequence *CYP2C19* diplotype** *(blank = not required)* | **Comments** |
| --- | --- | --- | --- | --- |
| 001 | *1/*1 | *1/*1 |  |  |
| 002 | *1/*17 | *1/*17 |  |  |
| 003 | *1/*17 | *1/*17 |  |  |
| 004 | *8/*17 | *8/*17 |  |  |
| 005 | Test fail | *1/*1 |  | Further sample not available |
| 006 | *1/*2 | *1/*2 |  |  |
| 007 | *1/*2 | *1/*2 |  |  |
| 008 | *1/*2 | *1/*2 |  |  |
| 009 | *1/*1 | *1/*1 |  |  |
| 010 | *1/*1 | *1/*1 |  |  |
| 011 | *2/*2 | *2/*2 |  |  |
| 012 | *1/*17 | *1/*17 |  |  |
| 013 | *1/*2 | *1/*2 |  |  |
| 014 | *1/*1 | *1/*1 |  |  |
| 015 | *17/*17 | *17/*17 |  |  |
| 016 | *1/*1 | *1/*1 |  |  |
| 017 | *17/*17 | *17/*17 |  |  |
| 018 | *1/*1 | *1/*1 |  |  |
| 019 | *17/*17 | *17/*17 |  |  |
| 020 | *1/*17 | *1/*17 |  |  |
| 021 | *1/*1 | SNP FAIL | *1/*1 |  |
| 022 | *1/*1 | *1/*1 |  |  |
| 023 | *1/*17 | *1/*17 |  |  |
| 024 | *1/*1 | *1/*1 |  |  |
| 025 | *1/*17 | *1/*17 |  |  |
| 026 | *1/*17 | *1/*17 |  |  |
| 027 | *1/*17 | *1/*17 |  |  |
| 028 | *2/*2 | *2/*2 |  |  |
| 029 | *2/*2 | *2/*2 |  |  |
| 030 | *1/*1 | *1/*1 |  |  |
| 031 | *1/*1 | *1/*1 |  |  |
| 032 | *1/*2 | *1/*2 |  |  |
| 033 | *1/*1 | *1/*1 |  |  |
| 034 | *2/*17 | *1/*17 | *2/*17 |  |
| 035 | *1/*1 | *1/*1 |  |  |
| 036 | *1/*17 | *1/*17 |  |  |
| 037 | *1/*2 | *1/*2 |  |  |
| 038 | *1/*2 | *1/*2 |  |  |
| 039 | *1/*2 | *1/*2 |  |  |
| 040 | *1/*2 | *1/*2 |  |  |
| 041 | *1/*1 | *1/*1 |  |  |
| 042 | *1/*1 | *1/*17 | *1/*1 |  |
| 043 | *1/*2 | *1/*2 |  |  |
| 044 | *1/*35 | *1/*1 | *1/*35 |  |
| 045 | *1/*17 | *1/*17 |  |  |
| 046 | *1/*17 | *1/*17 |  |  |
| 047 | *1/*17 | *1/*17 |  |  |
| 048 | *2/*17 | SNP FAIL | SNP FAIL | *2/*17 via Agena on a Buccal Sample |
| 049 | *1/*17 | *1/*17 |  |  |
| 050 | *1/*17 | *1/*17 |  |  |
| 051 | *1/*1 | *1/*1 |  |  |
| 052 | *1/*2 | *1/*2 |  |  |
| 053 | *1/*17 | *1/*17 |  |  |
| 054 | *1/*1 | *1/*1 |  |  |
| 055 | *1/*1 | SNP FAIL | *1/*1 |  |
| 056 | *17/*17 | *17/*17 |  |  |
| 057 | *1/*2 | *1/*2 |  |  |
| 058 | *1/*1 | *1/*1 |  |  |
| 059 | *2/*17 | *2/*17 |  |  |
| 060 | *1/*17 | *1/*17 |  |  |
| 061 | *17/*35 | *1/*17 | *17/*35 |  |
| 062 | *1/*1 | *1/*1 |  |  |
| 063 | *1/*2 | *1/*2 |  |  |
| 064 | *2/*17 | *2/*17 |  |  |
| 065 | *1/*8 | SNP FAIL | *1/*8 |  |
| 066 | *1/*17 | *1/*17 |  |  |
| 067 | *1/*1 | *1/*1 |  |  |
| 068 | *1/*2 | *1/*2 |  |  |
| 069 | *1/*1 | *1/*1 |  |  |
| 070 | *2/*2 | *2/*2 |  |  |
| 071 | *17/*17 | *17/*17 |  |  |
| 072 | *1/*17 | *1/*17 |  |  |
| 073 | *1/*2 | *1/*2 |  |  |
| 074 | *1/*1 | *1/*1 |  |  |
| 075 | *17/*17 | *1/*17 | *17/*17 |  |
| 076 | *2/*17 | *2/*17 |  |  |
| 077 | *1/*1 | *1/*1 |  |  |
| 078 | *1/*1 | *1/*1 |  |  |
| 079 | *1/*1 | *1/*1 |  |  |
| 080 | *1/*17 | *1/*17 |  |  |
| 081 | *1/*17 | *1/*17 |  |  |
| 082 | *1/*1 | *1/*1 |  |  |
| 083 | *1/*17 | *1/*17 |  |  |
| 084 | *1/*17 | *1/*17 |  |  |
| 085 | *1/*17 | *1/*17 |  |  |
| 086 | *1/*1 | *1/*1 |  |  |
| 087 | No human DNA found | *17/*2 |  | Resample from donor not available. |
| 088 | *1/*17 | *1/*17 |  |  |
| 089 | *1/*1 | *1/*1 |  |  |
| 090 | *2/*17 | *2/*17 |  |  |
| 091 | *1/*1 | *1/*1 |  |  |
| 092 | *17/*17 | *1/*17 | *17/*17 |  |
| 093 | *1/*17 | *1/*17 |  |  |
| 094 | *1/*17 | *1/*17 |  |  |
| 095 | *1/*1 | *1/*1 |  |  |
| 096 | *1/*2 | SNP FAIL | *1/*2 |  |
| 097 | *1/*1 | *1/*1 |  |  |
| 098 | *1/*1 | *1/*1 |  |  |
| 099 | *1/*1 | SNP FAIL | *1/*1 |  |
| 100 | *2/*2 | *2/*2 |  |  |
| 101 | *1/*1 | *1/*1 |  |  |
| 102 | *1/*17 | *1/*17 |  |  |
| 103 | *1/*17 | *1/*17 |  |  |
| 104 | *1/*17 | *1/*17 |  |  |
| 105 | *2/*2 | *2/*2 |  |  |
| 106 | *1/*1 | *1/*1 |  |  |
| 107 | *1/*2 | *1/*2 |  |  |
| 108 | *1/*17 | *1/*17 |  |  |
| 109 | *1/*1 | *1/*1 |  |  |
| 110 | *2/*17 | *2/*17 |  |  |
| 111 | *1/*17 | *1/*17 |  |  |
| 112 | *1/*1 | *1/*1 |  |  |
| 113 | *1/*17 | *1/*17 |  |  |
| 114 | *1/*2 | *1/*2 |  |  |
| 115 | *1/*1 | *1/*1 |  |  |
| 116 | *1/*1 | *1/*1 |  |  |
| 117 | *17/*17 | *17/*17 |  |  |
| 118 | *1/*1 | *1/*1 |  |  |
| 119 | *1/*2 | *1/*2 |  |  |
| 120 | *2/*17 | *2/*17 |  |  |
| 121 | *1/*17 | *1/*17 |  |  |
| 122 | *1/*1 | *1/*1 |  |  |
| 123 | *1/*1 | *1/*1 |  |  |
| 124 | *1/*17 | *1/*17 |  |  |
| 125 | *1/*1 | *1/*1 |  |  |
| 126 | *1/*17 | *1/*17 |  |  |
| 127 | *1/*2 | *1/*2 |  |  |
| 128 | *1/*17 | *1/*17 |  |  |
| 129 | *1/*1 | *1/*1 |  |  |
| 130 | *1/*2 | *1/*2 |  |  |
| 131 | *1/*17 | *1/*17 |  |  |
| 132 | *2/*17 | *2/*17 |  |  |
| 133 | *1/*1 | *1/*1 |  |  |
| 134 | *1/*2 | *1/*2 |  |  |
| 135 | *1/*17 | *1/*17 |  |  |
| 136 | *17/*17 | *17/*17 |  |  |
| 137 | *1/*1 | *1/*1 |  |  |
| 138 | *1/*2 | *1/*2 |  |  |
| 139 | *1/*17 | *1/*17 |  |  |
| 140 | *1/*17 | *1/*17 |  |  |
| 141 | *1/*1 | *1/*1 |  |  |
| 142 | *1/*17 | *1/*17 |  |  |
| 143 | *1/*3 | *1/*3 |  |  |
| 144 | *1/*1 | *1/*1 |  |  |
| 145 | *1/*2 | *1/*2 |  |  |
| 146 | *1/*1 | *1/*1 |  |  |
| 147 | *1/*2 | *1/*2 |  |  |
| 148 | *1/*1 | *1/*1 |  |  |
| 149 | *1/*2 | *1/*2 |  |  |
| 150 | *1/*1 | *1/*1 |  |  |
| 151 | *2/*17 | *2/*17 |  |  |
| 152 | *1/*1 | *1/*1 |  |  |
| 153 | *1/*17 | *1/*17 |  |  |
| 154 | *1/*17 | *1/*17 |  |  |
| 155 | *17/*17 | *17/*17 |  |  |
| 156 | *1/*17 | *1/*17 |  |  |
| 157 | *1/*1 | *1/*1 |  |  |
| 158 | *1/*1 | *1/*1 |  |  |
| 159 | *1/*1 | *1/*1 |  |  |
| 160 | *1/*17 | *1/*17 |  |  |
| 161 | *1/*1 | *1/*1 |  |  |
| 162 | *1/*17 | *1/*1 | *1/*17 | Buccal sample sequenced. Patient had HSCT. |
| 163 | *1/*35 | *1/*1 | *1/*35 |  |
| 164 | *1/*1 | *1/*1 |  |  |
| 165 | *1/*1 | *1/*1 |  |  |
| 166 | *1/*17 | *1/*17 |  |  |
| 167 | *1/*17 | *1/*17 |  |  |
| 168 | *1/*17 | *1/*17 |  |  |
| 169 | *1/*1 | *1/*1 |  |  |
| 170 | *1/*1 | *1/*1 |  |  |
| 171 | *2/*17 | *2/*17 |  |  |
| 172 | *1/*1 | *1/*1 |  |  |
| 173 | *1/*1 | *1/*1 |  |  |
| 174 | *1/*1 | *1/*1 |  |  |
| 175 | *1/*17 | *1/*17 |  |  |
| 176 | *2/*17 | *2/*17 |  |  |
| 177 | *1/*17 | *1/*17 |  |  |
| 178 | *1/*1 | *1/*1 |  |  |
| 179 | *1/*2 | *1/*2 |  |  |
| 180 | *1/*1 | *1/*1 |  |  |
| 181 | *17/*17 | *17/*17 |  |  |
| 182 | *1/*1 | *1/*1 |  |  |
| 183 | *1/*1 | *1/*1 |  |  |
| 184 | *1/*17 | *1/*17 |  |  |
| 185 | *1/*1 | *1/*1 |  |  |
| 186 | *8/*17 | *8/*17 |  |  |
| 187 | *1/*2 | *1/*2 |  |  |
| 188 | *1/*2 | *1/*2 |  |  |
| 189 | *1/*1 | *1/*1 |  |  |
| 190 | *1/*1 | *1/*1 |  |  |
| 191 | *1/*17 | *1/*17 |  |  |
| 192 | *1/*17 | *1/*17 |  |  |
| 193 | *1/*1 | *1/*1 |  |  |
| 194 | *1/*17 | *1/*17 |  |  |
| 195 | *1/*1 | *1/*1 |  |  |
| 196 | *2/*17 | *2/*17 |  |  |
| 197 | *17/*17 | *17/*17 |  |  |
| 198 | *1/*1 | *1/*1 |  |  |
| 199 | *2/*17 | *2/*17 |  |  |
| 200 | *1/*17 | *1/*17 |  |  |
| 201 | *2/*17 | *2/*17 |  |  |
| 202 | *2/*17 | *17/*2 |  |  |
| 203 | *4/*17 | *4/*17 |  |  |
| 204 | *2/*17 | *2/*17 |  |  |

**Supplemental Table S2. Complete Results.** HSCT = Hematopoietic stem cell transplant. SNP = Single Nucleotide Polymorphism. Where there is a “SNP Fail” it was not possible to identify the *CYP2C19* diplotype.
